# Vitamin D_3_ deficiency and osteopenia in spastic paraplegia type 5 indicate impaired bone homeostasis

**DOI:** 10.1038/s41598-024-53057-5

**Published:** 2024-03-27

**Authors:** Sabrina Ehnert, Stefan Hauser, Holger Hengel, Philip Höflinger, Rebecca Schüle, Tobias Lindig, Jonathan Baets, Tine Deconinck, Peter de Jonghe, Tina Histing, Andreas K. Nüssler, Ludger Schöls, Tim W. Rattay

**Affiliations:** 1 Siegfried Weller Research Institute at the BG Unfallklinik Tübingen, Department of Trauma and Reconstructive Surgery, University of Tübingen, Schnarrenbergstr. 95, 72076 Tübingen, Germany; 2 German Center of Neurodegenerative Diseases (DZNE), 72076 Tübingen, Germany; 3 Department of Neurodegenerative Disease, Hertie-Institute for Clinical Brain Research, and Center for Neurology, University of Tübingen, Hoppe-Seyler-Straße 3, 72076 Tübingen, Germany; 4 Department of Diagnostic and Interventional Neuroradiology, University Hospital Tübingen, 72076 Tübingen, Germany; 5 Neurogenetics Group, Center for Molecular Neurology, VIB, 2610 Antwerp, Belgium; 6 Department of Neurology, Antwerp University Hospital, 2610 Antwerp, Belgium; 7 Laboratory of Neuromuscular Pathology, Institute Born-Bunge, University of Antwerp, 2610 Antwerp, Belgium; 8 BG Unfallklinik Tübingen, Department of Trauma and Reconstructive Surgery, University of Tübingen, Schnarrenbergstr. 95, 72076 Tübingen, Germany; 9 Center for Rare Diseases (ZSE), University of Tübingen, Tübingen, Germany; 10 Center for Rare Diseases (ZSE), University of Schleswig Holstein, Kiel, Germany; 11 Department of Neurology, Christian-Albrechts University, Kiel, Germany

**Keywords:** Clinical genetics, Genetics, Molecular biology, Neuroscience, Biomarkers, Endocrinology, Medical research, Molecular medicine, Neurology

## Abstract

Hereditary spastic paraplegia type 5 (SPG5) is an autosomal recessively inherited movement disorder characterized by progressive spastic gait disturbance and afferent ataxia. SPG5 is caused by bi-allelic loss of function mutations in *CYP7B1* resulting in accumulation of the oxysterols 25-hydroxycholesterol and 27-hydroxycholesterol in serum and cerebrospinal fluid of SPG5 patients. An effect of 27- hydroxycholesterol via the estrogen and liver X receptors was previously shown on bone homeostasis. This study analyzed bone homeostasis and osteopenia in 14 SPG5 patients as a non-motor feature leading to a potential increased risk for bone fractures. T-Scores in CT bone density measurements were reduced, indicating osteopenia in SPG5 patients. Further, we analyzed various metabolites of bone homeostasis by ELISA in serum samples of these patients. We identified a lack of vitamin D_3_ metabolites (Calcidiol and Calcitriol), an increase in Sclerostin as a bone formation/mineralization inhibiting factor, and a decrease in cross-linked N-telopeptide of type I collagen (NTX), a marker indicating reduced bone resorption. As statin treatment has been found to lower oxysterol levels, we evaluated its effect in samples of the STOP-SPG5 trial and found atorvastatin to normalize the increased sclerostin levels. In summary, our study identified osteopenia as a non-motor feature in SPG5 and suggests the need for vitamin D_3_ substitution in SPG5 patients. Sclerostin may be considered a therapeutic target and biomarker in upcoming therapeutical trials in SPG5.

## Introduction

**Figure 1:**
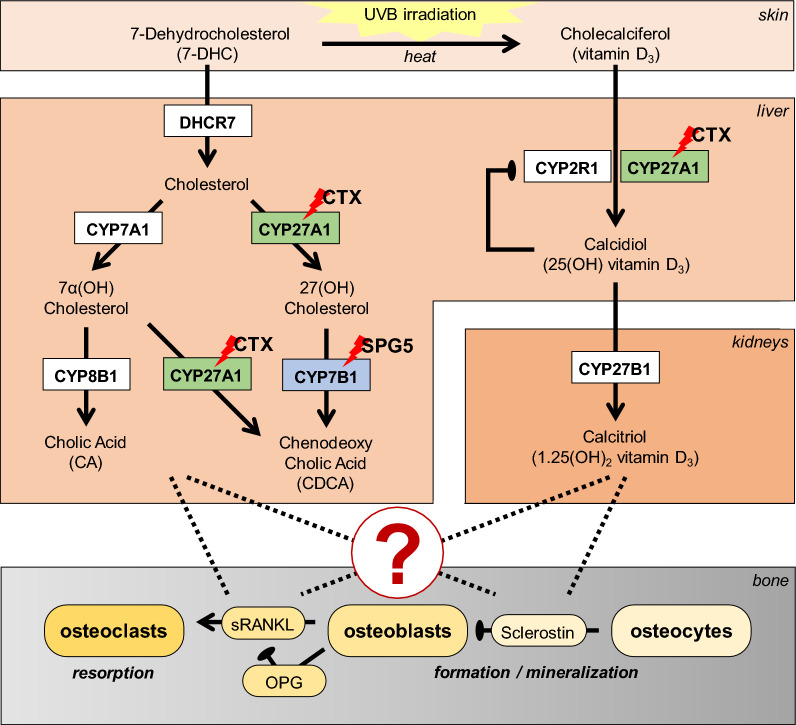
This figure illustrates the vitamin D_3_ metabolism and the involved organs (skin, liver, kidney, and bone). Both enzymes (highlighted by red lightening) are affected in the liver. For Hereditary Spastic Paraplegia Type 5 (SPG5) there is a defect in *CYP7B1* and in Cerebrotendinous Xanthomatosis (CTX) in *CYP27A1*. Critical pathways of bone homeostasis are shown on the bottom. *OPG* osteoprotegerin (a sRANKL antagonist), *sRANKL* soluble receptor activator of nuclear factor-κB ligand (sRANKL).

Hereditary spastic paraplegia (HSP)^1^ is a group of rare movement disorders characterized by progressive lower limb spasticity and weakness. Clinically, HSPs treatment options are limited to symptomatic treatment options in general^2,3^. HSP subtype 5 (SPG5) is caused by bi-allelic loss of function mutations in *CYP7B1* coding for the oxysterol-7α-hydroxylase. *CYP7B1* is involved in synthesizing primary bile acids from cholesterol via the "acidic" pathway in the liver. Side-chain oxidation of cholesterol by *CYP27A1* forms the oxysterols 25-hydroxycholesterol (25-OHC) and 27-hydroxycholesterol (27-OHC). Both 25-OHC and 27-OHC are physiologically degraded by 7α-hydroxylation via *CYP7B1* with high efficacy (compare Fig. 1). Loss of *CYP7B1* function in SPG5 leads to vastly elevated levels of oxysterols in SPG5 patients^4^, both in serum and cerebrospinal fluid (CSF). In contrast to cholesterol, oxysterols can pass the blood–brain barrier and were shown to be neurotoxic in neuronal cells in concentrations close to levels found in SPG5 patients^4^. Therefore, therapeutic approaches aimed to reduce oxysterol levels in SPG5 patients by diminishing its precursor cholesterol with lipid-lowering drugs like statins or ezetimibe^5,6^. For more efficient reduction of oxysterols, mRNA-mediated enzyme replacement has been tested in *Cyp7b1*^-/-^ knockout mice^7^.

Conversely, decreased levels of 27-OHC and its metabolites are the metabolic hallmarks of cerebrotendinous xanthomatosis (CTX) caused by loss of function mutations in *CYP27A1*^8^. CTX typically manifests with diarrhea in childhood due to lack of bile acids and juvenile cataracts due to deposits of abnormal lipids in the eye followed by neurological manifestation in adulthood with ataxia, spasticity, and dementia due to accumulation of abnormal lipids and lack of 27-OHC in the brain^8^. Of interest, *CYP27A1* also hydroxylates vitamin D3, an essential modification to enable its physiological activity^9^ (see Fig. 1).

Among many systemic effects (reviewed in^5^), oxysterols regulate bone density^10,11^. In this study, we measured bone density in SPG5 patients and assessed serum samples of SPG5 and CTX patients to analyze levels of vitamin D_3_ and its metabolites as well as regulators of osteoblasts and osteoclasts.

## Materials and methods

### Cohort

We included all 14 SPG5 patients from the STOP-SPG5 study^5^ and assessed serum samples from the baseline visit (N = 14) and follow-up samples of both arms (verum = atorvastatin and placebo; N = 7, each) at the end of the study after nine weeks. In addition, we recruited age- (± 2 years) and gender-matched healthy controls and sampled serum at baseline and after nine weeks at a similar period of the year (12/2019–02/2020) as the STOP-SPG5 study to control for seasonal effects (details for this cohort be found in Table 1). All those samples collected in the morning (between 8 and 10 am) and fasting (last food intake prior to 22:00). Further, serum samples of CTX patients (N = 5), as well as gender and age-matched healthy controls, were derived from the Neuro-Biobank at the Hertie Institute for Clinical Brain Research at the University of Tübingen. Those samples were not routinely fasting or all sampled at the same time.

**Table 1:** Cohort charateristiscs:

	Patients				Matched-controls	
SPG5		All cases n = 14	Verum n = 7	Placebo n = 7		n = 14
	Gender (f/m)	8/6	3/4	5/2	Gender (f/m)	8/6
	Age	38.5 (19–47)	44 (19–50)	35 (19.45)	Age	37.0 [18–49]
**CTX**		n = 5				n = 5
	Gender (f/m)	4/1			Gender (f/m)	4/1
	Age	54 [29–61]			Age	46 [23–57]

Age (years): median [range: minimut to maximum].

Published by Schöls et al.^5^.

*f* females, *m* males.

### Ethics approval

The STOP-SPG5 study^5^ was carried out in compliance with the Helsinki Declaration and approved by the Institutional Review Board of the University of Tübingen reference number: 247/2015 and the national regulatory institution (Bundesamt für Arzneimittel und Medizinprodukte—BfArM) and is registered as EudraCT 2015-000978-35. Serum samples from healthy controls were provided by the Hertie Institute for Clinical Brain Research following the regulations of the Neuro-Biobank and vote 199/2011BO1 of the Institutional Review Board of the University of Tübingen. Informed consent was obtained from all individuals.

### Enzyme-linked immunosorbent assay (ELISA)

Target proteins in serum samples were quantified with the help of ELISA kits, performed as indicated by the manufacturer. An overview is given in Table 2. Each sample was measured as duplicate. Photometric detection of the ELISA signals was performed with the OMEGA Plate-reader (BMG Labtech, Orthenberg, Germany). Measurements were repeated when the measurement exceeded the standard curve or duplicates were inconsistent. Furthermore, the measurement of 2 internal controls (high and a low concentration of the target protein) was performed as an additional quality control.

**Table 2:** Enzyme linked immunosorbent assay (ELISA).

Target	Function	ELISA kit		Dilution factor
		Order #	Company	
25(OH) vit. D_3_	25OH vitamin D_3_	AC-57DF1	IDS	–
1,25(OH)_2_ vit. D_3_	1,25(OH)_2_ vitamin D_3_	KAP1921	DIA Source	2
BAP	Osteoblast activity	AC-20F1	IDS	–
PINP	Collagen synthesis/bone formation	8003	TecoMedical	10
TRAP5b	Osteoclast activity	SB-TR201A	IDS	–
NTX	Collagen degradation/bone resorption	E-EL-H0836	Elabscience	10
sRANKL	Favors osteoclastogenesis	900-K142	Peprotech	6
OPG	Inhibitor for sRANKL	ABIN411341	Antibodies-online	10
Sclerostin	Inhibitor for Wnt signaling and bone mineralization	ABIN2703466	Antibodies-online	5

### Bone density measurements

Quantitative computer tomography (qCT) was performed to measure bone density covering lumbar vertebrae 1–3 (85 kV, 125 mA) and analyzed, referencing a calcium hydroxylapatite (CaHA) phantom.

### Statistics

Results are represented as box blots (Box and Whiskers—Tukey to visualize outliers) or scatter diagrams (median ± interquatile range with individual data points). The number of donors (N) and technical replicates (n) is given in the figure legends. The baseline levels (SPG5 vs. CO and CTX vs. CO) were compared using the non-parametric Wilcoxon matched-pairs signed-rank test (2-tailed). The treatment groups (placebo vs. verum vs. controls) were exploratory compared using the non-parametric Friedman test (matched pairs) followed by the two-stage linear step-up procedure of Benjamini, Krieger, and Yekutieli to correct for multiple comparisons. The text summarizes data as mean ± SEM (95% confidence interval). A Spearman correlation matrix was generated (Supplementary Fig. S1) to identify possible correlations between the different serum parameters. Statistical analysis was performed using the GraphPad Prism Software (Version 8.0, El Camino Real, USA). A *p* < 0.05 at an α = 0.05 was taken as the minimum significance level.

## Results

### Clinical findings

In four of the fourteen patients of the STOP-SPG5^5^ study a developmental hip dysplasia and in one patient a bilateral aseptic femoral bone necrosis in childhood was observed. Osteoporosis was only known in one female prior to our bone density measurements, with no previous osteoporosis diagnostic procedures being performed in any patient. After traumatic rib serial fractures at 45 years, she was diagnosed with osteoporosis and consequently treated with cholecalciferol. Two patients reported previous sports-associated traumatic bone fractures at the ankle and metatarsal. Further risk factors included smoking in 2/14 (15%) cases; there was no history of early bone fractures in parents as obligatory heterozygous *CYP7B1* mutation carriers.

### qCT bone density reveals osteopenia

qCT-bone density measurements were available for three patients only as part of a pilot measurement. For age- and -gender-specific cutoffs, osteopenia was found in all three patients (T- score classification of the WHO, osteopenia [− 1 to − 2.5]). The mean trabecular bone density reached from 98.5 to 121.8 mg CaHA/ml, with a cortical bone density between 264.3 and 301.3 mg CaHA/ml. Z-Scores reached from − 0.23 to − 0.99 and T-scores from − 1.36 to − 2.20. In detail the finding were: one 49 years old male (T: − 2.11; Z: − 0.23), one 40 years old female (T: − 1.36; Z: − 0.99), and one 49 years old female (T: − 2.20; Z: − 0.82).

### Decreased levels of vitamin D_3_ metabolites in blood of SPG5 patients

**Figure 2:**
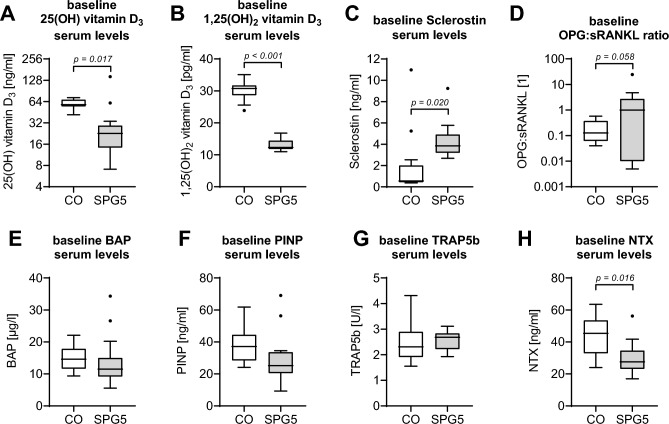
Baseline levels of serum markers assessed by ELISA in hereditary spastic paraplegia type 5 (SPG5) patients and matched controls (CO) (N = 14, per group). For displaying vitamin D_3_ metabolism, basal levels of (**A**) 25(OH) vitamin D_3_ and (**B**) 1,25(OH)_2_ vitamin D_3_ were determined. As regulators of osteoblast and osteoclast function, basal levels of (**C**) sclerostin, as well as (**D**) the ratio of soluble receptor activator of nuclear factor-κB ligand (sRANKL), and its antagonist osteoprotegerin (OPG), were determined. Osteoblast function is displayed by (**E**) bone alkaline phosphatase (BAP) and associated collagen formation by (**F**) N-terminal propeptide of human procollagen type I (PINP) levels. Osteoclast function is displayed by (**G**) tartrate-resistant acidic phosphatase 5b (TRAP5b) and associated collagen degradation by (**H**) cross-linked N-telopeptide of type I collagen (NTX). Each sample was measured in duplicates (n = 2). Both groups were compared using the non-parametric Wilcoxon matched-pairs signed-rank test (2-tailed), with *p* < 0.1 being specified in the figure.

Serum of SPG5 patients and matched controls (N = 14, per group) were analyzed for the presence of 25(OH) vitamin D_3_ (Calcidiol) and 1,25(OH)_2_ vitamin D_3_ (Calcitriol) by ELISA. Basal Calcidiol levels were significantly lower in SPG5 patients (31.7 ± 9.4 ng/ml) than in matched controls (59.2 ± 2.2 ng/ml; *p* = 0.017—Fig. 2A). Calcidiol levels were in 11/14 (78.6%) of SPG5 patients below the recommended threshold of 30 ng/ml (international position statement^12^) compared to none in the healthy controls. Even more pronounced was the difference in basal Calcitriol levels (SPG5 patients: 13.1 ± 0.5 pg/ml vs. matched controls: 30.0 ± 0.8 pg/ml; *p* < 0.001—Fig. 2B). As expected, a strong positive correlation between Calcidiol levels and Calcitriol levels was observed (r = 0.56; *p* = 0.002).

### High levels of sclerostin in blood of SPG5 patients

Serum levels of sclerostin, soluble receptor activator of nuclear factor-κB ligand (sRANKL), and its antagonist osteoprotegerin (OPG) were measured as regulators of osteogenesis and osteoclastogenesis. Sclerostin levels were significantly higher in serum of SPG5 patients (4.3 ± 0.5 ng/ml) than in matched controls (1.9 ± 0.8 ng/ml; *p* = 0.020—Fig. 2C). Independent of the disease state, sclerostin levels showed a strong negative correlation to Calcitriol levels (r = − 0.60; *p* = 0.001) but not to Calcidiol levels in the investigated samples. The ratio of OPG to sRANKL shifted towards OPG in SPG5 patients (2.9 ± 1.7) when compared to matched controls (0.2 ± 0.1; *p* = 0.058—Fig. 2D). In contrast to sclerostin, OPG and sRANKL both showed a weak correlation with Calcidiol (OPG: r = − 0.44; *p* = 0.019 and sRANKL: r = 0.35; *p* = 0.066) but not with Calcitriol.

Osteoblast function was determined by serum levels of bone alkaline phosphatase (BAP). Basal BAP serum levels were comparable between SPG5 patients (13.7 ± 2.2 µg/l) and matched controls (14.7 ± 1.2 µg/l; *p* > 0.05—Fig. 2E). Associated collagen formation, displayed by N-terminal propeptide of human procollagen type I (PINP) levels, was slightly lower in SPG5 patients (29.2 ± 4.2 ng/ml) than in matched controls (37.7 ± 2.9 ng/ml; *p* > 0.05—Fig. 2F). Osteoclast function was determined by serum levels of tartrate-resistant acidic phosphatase 5b (TRAP5b). Basal TRAP5b levels were comparable between SPG5 patients (2.6 ± 0.1 U/l) and matched controls (2.4 ± 0.2 U/l; *p* > 0.05—Fig. 2G). Associated collagen degradation, displayed by cross-linked N-telopeptide of type I collagen (NTX), was lower in SPG5 patients (29.6 ± 2.7 ng/ml) than in matched controls (43.9 ± 3.4 ng/ml; *p* = 0.016—Fig. 2H). Interestingly, NTX levels did not correlate with TRAP5b, OPG, or sRANKL levels, but strongly correlated with Calcitriol levels (r = 0.53; *p* = 0.004) and sclerostin levels (r = − 0.59; *p* = 0.001), independent of the disease state.

### No significant effect of atorvastatin treatment on vitamin D3 metabolism in SPG5

**Figure 3:**
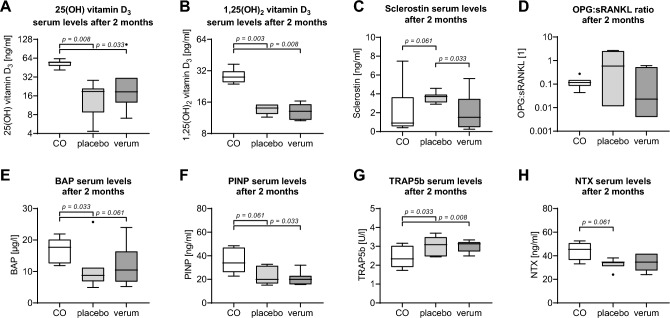
Serum markers in hereditary spastic paraplegia type 5 (SPG5) patients two months after treatment and matched controls. SPG5 patients were randomly assigned to receive treatment (verum) or placebo (N = 7 per group). After two months, serum levels of the specific markers were controlled (ELISA) in the SPG5 patients and matched controls. Markers analyzed were: (**A**) 25(OH) vitamin D_3_, (**B**) 1,25(OH)_2_ vitamin D_3_, (**C**) sclerostin, (**D**) the ratio of soluble receptor activator of nuclear factor-κB ligand (sRANKL), and its antagonist osteoprotegerin (OPG), (**E**) bone alkaline phosphatase (BAP), (**F**) N-terminal propeptide of human procollagen type I (PINP), (**G**) tartrate-resistant acidic phosphatase 5b (TRAP5b), and (**H**) Cross-linked N-telopeptide of type I collagen (NTX). Each sample was measured in duplicates (n = 2). The groups were compared by the non-parametric Friedman test (matched pairs) followed by the two-stage linear step-up procedure of Benjamini, Krieger, and Yekutieli to correct for multiple comparisons. All *p* < 0.1 are shown in the graphic.

After sampling for baseline levels, SPG5 patients were randomly assigned to receive atorvastatin (verum, N = 7) or placebo (N = 7) in a double-blinded study. After two months, serum levels of the vitamin D_3_ metabolites, Calcidiol, and Calcitriol were measured. Although Calcidiol levels seemed higher in the verum group (30.1 ± 13.0 ng/ml) than in the placebo group (16.7 ± 3.1 ng/ml; *p* = 0.593), Calcidiol levels were still significantly lower in SPG5 patients than in the matched controls (53.1 ± 2.5 ng/ml; *p* = 0.008 and *p* = 0.033, respectively—Fig. 3A). In Calcitriol as a downstream metabolite, no trend could be observed. In both the verum group (13.2 ± 0.9 pg/ml; *p* = 0.008) and the placebo group (13.7 ± 0.5 pg/ml; *p* = 0.003), Calcitriol levels were significantly lower than in matched controls (28.9 ± 1.8 pg/ml—Fig. 3B).

### Serum levels of sclerostin were significantly reduced in SPG5 patients by the treatment

Although no significant effect of atorvastatin treatment on vitamin D_3_ metabolism was observed, serum sclerostin levels were significantly reduced in the verum group (2.1 ± 0.7 ng/ml) compared to the placebo group (3.7 ± 0.2 ng/ml; *p* = 0.033), reaching levels comparable to matched controls (2.1 ± 1.0 ng/ml—Fig. 3C). A similar trend was observed for the ratio of OPG:sRANKL. In comparison to the placebo group (1.1 ± 0.4) the OPG:sRANKL ratio shifted towards sRANKL the verum group (0.2 ± 0.1; *p* = 0.109), reaching levels comparable to the matched controls (0.1 ± 0.0—Fig. 3D). This change could be mainly assigned to a strong increase in sRANKL by the verum treatment (placebo: 10.9 ± 6.5 ng/ml vs. verum 151.6 ± 74.6 ng/ml; *p* = 0.061).

In line with the decrease in sclerostin levels, BAP serum levels showed an increasing trend in the verum group (12.0 ± 2.4 µg/l) compared to the placebo group (10.8 ± 2.6 µg/l; *p* = 0.789) but did not reach levels of matched controls (16.9 ± 1.5 µg/l—Fig. 3E). In the case of PINP, no effect of the verum treatment could be observed. PINP levels of both groups (placebo: 22.3 ± 2.8 ng/ml; *p* = 0.061 vs. verum 21.1 ± 2.1 ng/ml; *p* = 0.033) remained significantly lower than in matched controls (36.6 ± 4.0 ng/ml—Fig. 3F). The levels of the osteoclast marker TRAP5b remained unaffected by the atorvastatin treatment. TRAP5b levels of both groups (placebo: 3.0 ± 0.2 U/l; *p* = 0.033 vs. verum 3.0 ± 0.1 U/l / *p* = 0.008) were significantly higher than TRAP5b levels in the matched controls (2.4 ± 0.2 ng/ml—Fig. 3G). In line with this observation, NTX levels in SPG5 patients were not changed by atorvastatin treatment (placebo: 32.6 ± 1.7 ng/ml vs. verum 33.1 ± 2.7 ng/ml; *p* = 0.593), and bone turnover remained higher in matched controls (43.7 ± 2.9 ng/ml; *p* = 0.061 and *p* = 0.181, respectively—Fig. 3H).

### Exploratory findings in Cerebrotendinous Xanthomatosis (CTX)

**Figure 4:**
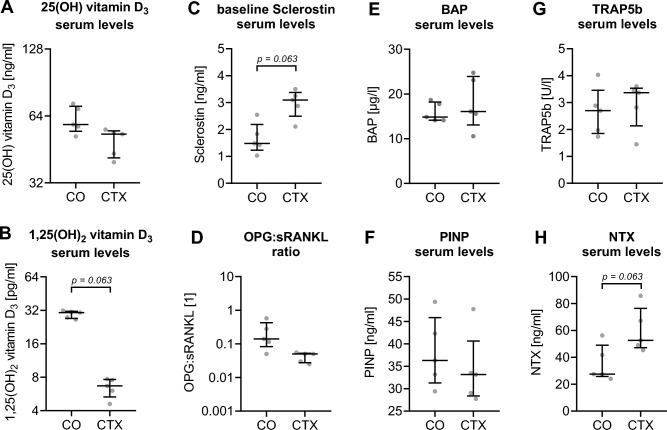
Serum markers in Cerebrotendinous xanthomatosis (CTX) patients and matched controls (CO). Each sample was measured in duplicates (n = 2). Both groups were compared by non-parametric Wilcoxon matched-pairs signed-rank test (2-tailed), with all *p* < 0.1 being specified in the figure. Serum levels of specific markers for CTX patients and matched controls (N = 5 per group) were determined by ELISA: (**A**) 25(OH) vitamin D_3_, (**B**) 1,25(OH)_2_ vitamin D_3_, (**C**) sclerostin, (**D**) the ratio of soluble receptor activator of nuclear factor-κB ligand (sRANKL), and its antagonist osteoprotegerin (OPG), (**E**) bone alkaline phosphatase (BAP), (**F**) N-terminal propeptide of human procollagen type I (PINP), (**G**) tartrate-resistant acidic phosphatase 5b (TRAP5b), and (**H**) Cross-linked N-telopeptide of type I collagen (NTX).

Given the opposing metabolic profile in CTX compared to SPG5, we analyzed serum samples of CTX patients (N = 5) in an exploratory approach. In contrast to SPG5 patients' basal serum levels of the primary vitamin D_3_ metabolite, Calcidiol did not significantly differ in sera of CTX patients compared to matched controls (Fig. 4A). However, a strong but not significant reduction of Calcitriol levels was observed in CTX patients (6.5 ± 0.6 pg/ml) compared to matched controls (29.5 ± 1.0 pg/ml; *p* = 0.063 – Fig. 4B) and SPG5 patients (13.1 ± 0.5 pg/ml—Fig. 2B). Similar to SPG5 patients, sclerostin levels had a trend to be higher in CTX patients (3.0 ± 0.2 ng/ml; *p* = 0.063) when compared to matched controls (1.7 ± 0.3 ng/ml—Fig. 4C). The strong negative correlation between Calcitriol and sclerostin observed in SPG5 patients was confirmed in CTX patients and matched controls (r = − 0.71; *p* = 0.027). The observed shift in the ratio of OPG to sRANKL towards OPG in SPG5 patients was not observed in CTX patients. In CTX patients, the ratio tendentially shifted towards sRANKL (Fig. 4D). The ratio of OPG to sRANKL showed a weak correlation with Calcidiol and Calcitriol (both r = 0.63; *p* = 0.056), which is attributed to a negative correlation between sRANKL and the two vitamin D_3_ metabolites Calcidiol (r = − 0.64; *p* = 0.052) and Calcitriol (r = − 0.68; *p* = 0.035). Interestingly, the ratio of OPG to sRANKL showed a negative correlation with sclerostin levels in CTX patients (r = − 0.71; *p* = 0.027), which was not observed in SPG5 patients.

Basal levels were equivalent between CTX patients and matched controls for BAP (Fig. 4E), PINP (Fig. 4F) and TRAP5b (Fig. 4G). NTX had a trend (*p* = 0.063) to be higher in CTX patients (Fig. 4H). In CTX patients NTX levels showed a weak correlation with sRANKL levels (r = 0.72; *p* = 0.022). In contrast to SPG5 patients, CTX patients showed a positive correlation between NTX and sclerostin (r = 0.78; *p* = 0.011), and a weak negative correlation between NTX and Calcitriol (r = − 0.58; *p* = 0.085).

## Discussion

This study identified bone mineral density (BMD) by qCT measurements in SPG5 patients as osteopenia. According to the WHO criteria, these findings are associated with an increased risk for bone fractures. Despite various treatment approaches, there is still no cure for SPG5. The non-motor features are increasingly recognized^13–15^ and can be used for symptomatic treatment, which is the key to improve the quality of life of HSP patients. With a severe increase in serum level of oxysterols in SPG5 and a known negative regulatory effect of oxysterols on bone density^10,11^, we set out to study bone homeostasis and vitamin D_3_ metabolism in patients with SPG5. It is of high importance to recognize osteoporosis and abnormal vitamin D3 metabolism in SPG5 patients as it is easy to treat and likely to improve patients' quality of life. As a first piece of evidence for the clinical relevance of altered bone homeostasis in SPG5, we found developmental hip dysplasia and aseptic femoral bone necrosis in childhood to be frequent in our SPG5 cohort and added clinical evidence of impaired bone homeostasis by bone density measurements. As a likely cause, we identified a reduction of vitamin D_3_ metabolites Calcidiol (78.6% of cases) and Calcitriol. For calcitriol no international consensus range for healthy adults exists but a large French study (888 healthy adults^16^) identified 29 ng/ml as confidence interval cut-off, which would result in 100% of SPG5 being below this threshold (compared to 50% of the healthy controls (5/14)). Another lower limit of the normal range is 18 ng/ml for both males and females as reported by Pagana et al.^17^ which would result also in 100% abnormal reduced SPG5 serum levels compare to 0% in healthy controls. The reduction of both metabolites leads to less intestinal calcium resorption and an increase in parathyroid hormone secretion resulting in bone resorption via activation of osteoclastogenesis by sRANKL^18^. We found the OPG:sRANKL ratio increased in SPG5, indicating a down-regulation of bone resorption—also shown by a reduction of NTX levels in SPG5 despite unchanged TRAP5b levels. However, bone remineralization is also affected by an increase in sclerostin which is usually produced by osteocytes inhibiting osteoblasts. However, high sclerostin serum levels can also originate from the liver, as shown in chronic liver disease (CLD)^19^. In summary, we detected complex bone homeostasis changes in SPG5, which can potentially be targeted by anti-osteoporotic drugs like Calcidiol^20^, Calcitriol^21^, and anti-sclerostin-antibodies^22,23^. These effects could also be driven by the small sample size.

Taking advantage of biomaterial preserved from the STOP-SPG5 study, we were able to study the influence of atorvastatin on vitamin D_3_ metabolism and bone homeostasis. As shown, atorvastatin treatment reduces 25-OH (25-OHC) and 27-OH oxysterols (27-OHC) in serum^5^. The adverse regulatory effects of oxysterols on BMD are well established^10,11^, but the pathomechanism is not fully understood. Statins have been shown to increase or keep Cholecalciferol levels similar (reviewed by Gupta et al.^24^) despite reducing the educt 7-Dehydrocholesterol (7-DHC), which is transformed via UVB irradiation (see Fig. 1) to cholecalciferol (vitamin D_3_). Statin effects on bone homeostasis have been assessed earlier in observational studies; *e.g.* a Taiwanese study^25^ reported an osteoprotective effect of statins with a decreased risk for osteoporosis. A small number of randomized control trials also showed an increase in BMD due to statins^26^ which was confirmed in a meta-analysis of seven randomized control trials totaling approx. 28,000 participants^27^. We did not observe an increase in Calcidiol (a product of cholecalciferol) in SPG5 patients after atorvastatin treatment in the STOP-SPG5 trial, probably due to the short treatment period. In contrast, in a cross-sectional study by Thabit et al*.*^26^, long-term effects of 30 participants on atorvastatin were analyzed, showing a direct effect of atorvastatin on Calcidiol levels; after more than one year of continuous intake, atorvastatin increased Calcidiol levels.

Sclerostin levels were found to be increased in SPG5 patients and to decrease to almost control levels under therapy with atorvastatin; however, no further changes in biomarkers of bone-metabolism were detected. The current results are limited by the short treatment duration of 9 weeks in the STOP-SPG5 trial. It remains unclear if positive mineralization effects with a reduction of osteoblasts inhibition via sclerostin would occur after more extensive treatment. Given the positive effects of atorvastatin on BMD in general^27^, the effect of atorvastatin in SPG5 patients could be driven by direct effects rather than lowering oxysterols. It remains unclear if an increased BMD caused by statins reduces fractures^27^. Therefore, the clinical relevance of increased BMD remains unclear.

In contrast to SPG5, 27-OHC are reduced in serum and CSF in CTX^8^. In the very small CTX cohort there is a trend to higher 25-OHC levels in serum and CSF^8^. Therefore, we used this disease as an additional model with an opposing metabolic profile with regards to 27-OHC. We were able to gain an exciting insight with limited significance due to the small sample size and lack of matched blood sampling time points for CTX. In contrast to published data indicating CTX occurs without calcium metabolism disorder^28,29^ and suggesting that another enzyme can catalyze vitamin D 25-hydroxylation^30^, we could identify a pronounced reduction of calcitriol levels compared to controls. In addition, we found a trend of an increase in sclerostin serum levels in CTX similar as in SPG5 and an increase of NTX serum levels in CTX contrary to SPG5. These analyses missed an alpha threshold of 5% with *p* = 0.063, most likely due to the limited number of CTX samples. Our data provides first hints toward a different pattern of bone homeostasis changes in CTX and SPG5, particularly for bone resorption (increase in NTX). These results need to be reproduced in larger cohorts to further evaluate calcitriol deficiency in CTX patients.

## Conclusion

This study found osteopenia and vitamin D_3_-deficiency as clinically relevant non-motor features in SPG5 patients. Inhibiting bone formation via sclerostin in combination with decreased bone-resorption impacts bone quality beyond fracture probabilities, as indicated by the Z-scores of bone density measurements in SPG5 patients. Our data suggest to include the analysis of vitamin D_3_ metabolites and bone homeostasis biomarkers in diseases with dysregulated oxysterol metabolism like SPG5 and CTX. We therefore suggest to include bone density measurements as standard of care in SPG5 patiente possibly resulting in osteopenia treatment to protect patients from progressing osteopenia and decrease their risk of bone fractures. Upcoming long-term observational studies should include bone density measurements and ongoing treatments as secondary outcome measurements to further study the relevance in SPG5 patients.

### Supplementary Information

Supplementary Figure 1.

## Data Availability

The datasets used during the current study available from the first author on reasonable request.
