# Supplementary material for: Vitamin D3 deficiency and osteopenia in spastic paraplegia type 5 indicate impaired bone homeostasis
Source: Sci Rep. 2024 Mar 27;14:7335. doi: 10.1038/s41598-024-53057-5 (PMC10973513; doi:10.1038/s41598-024-53057-5)
Supplement: Supplementary file 1 — Supplementary Figure 1. [file 41598_2024_53057_MOESM1_ESM.docx]

### Supplementary Material

**Supplementary Figure 1** **Spearman correlation matrix**

**Supplementary Figure 1:** Spearman correlation matrix obtained from basal serum levels of SPG5 patients (N = 14 – upper panel) or CTX patients (N = 5 – lower panel) and matched controls (N = 14 or 5). Serum levels were determined as duplicates (n = 2). Given are Spearman r (r), 95% confidence interval (C.I.) of the Spearman r, as well as the *p*-value (*p*). Due to the small sample size in CTX patients, the confidence intervals are not provided.
